# Position paper of Brazilian Society of Head and Neck Surgery among competencies in Head and Neck Surgery teaching for general Surgery residents^☆^^☆☆^

**DOI:** 10.1016/j.bjorl.2026.101875

**Published:** 2026-07-22

**Authors:** Mario Augusto Ferrari de Castro, Rogério Aparecido Dedivitis, Fátima Cristina Mendes de Matos, Marianne Yumi Nakai, Leandro Luongo de Matos, Marco Aurélio Vamondes Kulcsar, Carlos Takahiro Chone, Luiz Paulo Kowalski, Julia Fonseca Ferreira da Silva, Thiago Yuuki Kuroiwa

**Affiliations:** a Faculdade Medicina da Universidade Metropolitana de Santos, Department of Otorhinolaryngology - Head and Neck Surgery, Santos, SP, Brazil; b Faculdade de Medicina da Universidade de São Paulo, Department of Head and Neck Surgery, São Paulo, SP, Brazil; c Universidade Estadual de Pernambuco, Department of Head and Neck Surgery, Recife, PE, Brazil; d Faculdade de Ciências Médicas da Santa Casa de São Paulo, Department of Head and Neck Surgery, São Paulo, SP, Brazil; e Universidade Estadual de Campinas (UNICAMP), Department of Otorhinolaryngology - Head and Neck Surgery, Campinas, SP, Brazil; f Universidade Metropolitana de Santos, Faculdade Medicina, Santos, SP, Brazil

**Keywords:** Medical education, Clinical competencies, Internship and residency, Delphi method

## Abstract

**Objectives:**

To standardize the necessary competencies for General Surgery residents related to Head and Neck Surgery.

**Methods:**

The Delphi process was employed in its sequential phases: the selection of an expert panel; a structured questionnaire containing a preliminary list of potential competencies; the electronic distribution of the questionnaire to the experts, with options to “maintain”, “remove”, or “modify” the competencies and to suggest the inclusion of new competencies; a second round indicating “agree” or “disagree” for each reformulated or new competency; and the final consensus.

**Results:**

Twenty-two competencies were established for General Surgery residents in the specialty of Head and Neck Surgery.

**Conclusion:**

General Surgery residents and trainees should be trained for topics and procedures frequently encountered in general clinical practice. The establishment of minimum competencies emerges as a guide in Medical Education, aligning teaching with the actual needs of clinical practice.

**Level of evidence:**

4.

## Highlights

• A consensus defined head and neck surgery competencies for general surgery residents.
• A Delphi methodology was used to achieve agreement on essential competencies.
• The competency framework standardized surgical education.
• This study contributes to curriculum development in general surgery programs.

## Introduction

Contemporary medical education faces significant challenges, primarily related to the effective integration of theoretical and practical clinical competencies that respond to the dynamic demands of the medical profession. Within this context, the establishment of minimum competencies emerges as a guide in Medical Education, aligning teaching with the actual needs of clinical practice. Competencies are tasks or responsibilities that learners must be able to perform independently upon reaching certain levels of proficiency.^1^^,^^2^

In the specific field of Head and Neck Surgery, the integration of competencies into the educational curriculum is crucial, given the high prevalence of a wide variety of benign and malignant conditions in this region. General Surgery residents must master a series of competencies that extend beyond theoretical medical knowledge. Therefore, knowledge and skills in the area of head and neck surgery are indispensable for residents in training.^3^

For General Surgery residents and trainees, the adapted competencies from Head and Neck Surgery promote a deeper understanding of the interrelationships between different surgical specialties, preparing them to handle cases that require an interdisciplinary approach. Furthermore, proficiency in head and neck surgical procedures can be vital in trauma and emergency situations, where rapid and effective surgical management of the airway may be essential.^4^

The teaching of Head and Neck Surgery in General Surgery Residency and Fellowship programs is quite heterogeneous and, consequently, the competencies required of trainees in this specialty also vary. Thus, the objective of this study is to formulate the standardization of necessary competencies for these residents/fellows related to Head and Neck Surgery.

## Methods

This is a qualitative cross-sectional study to determine the competencies related to Head and Neck Surgery for General Surgery residents at the end of their training period. Data were collected through electronic questionnaires. The questionnaires were sent and distributed online to professors and heads of residency training services in Head and Neck Surgery accredited by the Brazilian Society of Head and Neck Surgery. The responses received from the questionnaires were organized and evaluated using the Delphi methodology, seeking to reach consensus.

To define the core competencies required for general surgery residents within the domain of head and neck surgery, we employed a modified Delphi consensus approach.^5^ This systematic method has demonstrated robust reliability in establishing educational standards across surgical specialties through structured expert consultation.

The consensus-building exercise was initiated by convening a select cohort of head and neck surgery division chiefs affiliated with the Brazilian Society of Head and Neck Surgery. Selection criteria prioritized individuals with substantial contributions to surgical education, clinical expertise, and administrative leadership to ensure comprehensive representation of training perspectives across the national landscape.

Our protocol implemented a sequential multi-phase design beginning with the compilation of a preliminary competency framework derived from contemporary surgical education literature and national general surgery training guidelines. This initial competency inventory was subjected to expert evaluation through an electronic survey platform.

During the first consensus iteration, participants evaluated each proposed competency using a categorical assessment framework (maintain/remove/modify). For competencies designated for modification, experts provided specific revision suggestions. The survey design also facilitated the proposition of additional competencies perceived as absent from the original framework.

Following completion of the initial round, comprehensive data analysis was performed to identify competencies achieving maintenance consensus, those recommended for elimination, and elements requiring refinement based on expert feedback. This analysis informed the development of a revised competency framework incorporating all suggested modifications and newly proposed competencies.

The second consensus iteration presented the refined framework to the expert panel using a binary response format (agree/disagree) for each competency with provision for supplementary qualitative commentary. This dichotomous assessment methodology is recognized as particularly effective in final consensus determination following initial exploratory rounds.

Definitive consensus was established at a threshold of 85% agreement following the second round, a deliberately stringent criterion to ensure that only unequivocally essential competencies were incorporated into the final curriculum framework for general surgery residents.

Throughout the entire process, participant anonymity was meticulously maintained ‒ a methodological cornerstone of Delphi technique that significantly mitigates confirmation bias, conformity pressure, and status-based influence in consensus formation. The research protocol received approval from the institutional ethics review board prior to implementation.

The entire process was conducted with anonymity among participants, a fundamental characteristic of the Delphi methodology that minimizes the influence of hierarchical or political factors in decisions.^6^

## Results

The investigation involved a panel of 25 specialists in Head and Neck Surgery who contributed throughout the entire Delphi methodology process. During the initial phase, 22 potential competencies were presented for evaluation. From these, 12 received unanimous endorsement, while 10 required revisions based on expert feedback. Additionally, participants recommended the removal of specific items and proposed three additional competencies not previously considered. In the subsequent evaluation phase, 19 competencies achieved complete consensus (100% agreement), while three received strong support with 95% agreement, establishing a final framework of 22 essential competencies for General Surgery residents, as detailed in Table 1. Table 2 shows the agreement results in the both consensus.

**Table 1:** Competencies of general surgery residents in head and neck surgery.

Nº	Competency
1	Act with ethics and professionalism
2	Communicate gently, effectively, and empathetically with patients, family members, and other professionals
3	Work appropriately in a team
4	Conduct thorough medical history for patients with head and neck conditions
5	Perform physical examination of patients
6	Identify the main risk factors for head and neck cancer
7	Identify the most prevalent head and neck conditions
8	Identify conditions requiring biopsy
9	Perform postoperative dressing changes
10	Remove surgical drains
11	Change tracheostomy tubes
12	Perform closure of surgical incisions
13	Evaluate and investigate patients with head and neck conditions
14	Recognize and indicate appropriate investigation for thyroid gland disorders
15	Evaluate, investigate, and develop an action plan for patients with acute airway obstruction
16	Evaluate, investigate, and operate on small cutaneous and subcutaneous lesions
17	Evaluate, investigate, and perform deep cervical drainage in patients with cervical abscess
18	Evaluate, investigate, and biopsy patients with suspicious cervical lymphadenopathy
19	Perform tracheostomy in intubated patients
20	Perform tracheostomy and/or cricothyroidotomy in patients with acute airway obstruction
21	Participate in the coordination, organization, and execution of surgical days
22	Participate in educational or administrative activities

**Table 2:** Agreement data for the competencies.

Competency	1st Row	2nd Row
1	100% agreement	100% agreement
2	95% agreement	100% agreement
3	100% agreement	100% agreement
4	100% agreement	100% agreement
5	100% agreement	100% agreement
6	100% agreement	100% agreement
7	100% agreement	100% agreement
8	100% agreement	100% agreement
9	95% agreement	100% agreement
10	95% agreement	100% agreement
11	95% agreement	95% agreement
12	100% agreement	100% agreement
13	100% agreement	100% agreement
14	100% agreement	100% agreement
15	100% agreement	100% agreement
16	Inclusion suggestion	100% agreement
17	55% agreement	100% agreement
18	95% agreement	95% agreement
19	100% agreement	100% agreement
20	95% agreement	100% agreement
21	Inclusion suggestion	100% agreement
22	100% agreement	100% agreement

## Discussion

The competency level of residents and fellows graduating from General Surgery programs requires that they be able to demonstrate basic competencies in practice, which means having professional knowledge, skills, and behaviors and knowing how to properly handle problems involving high levels of complexity.

Most head and neck tumors are diagnosed at advanced stages, due to patient and healthcare factors. Many patients experience a relative delay in diagnosis and treatment because they require multiple consultations before reaching a specialist. Furthermore, some general healthcare professionals fail to recognize certain signs and symptoms as suggestive of malignancy.^7^ Therefore, practical teaching activities should include proper loco-regional examination and training in recognizing risk factors for malignancy among patients with habits such as smoking, symptoms like persistent dysphonia, and signs such as oral ulcers that do not heal for more than three weeks.

Another situation to which a general surgeon must be aware is airway obstruction, a potentially life-threatening complication of head and neck cancer, worsened by potential the significant anatomic distortions. The most urgent goal of treatment in patients with severe obstruction is securing the airway. In the unsecured airway, obstruction may result from intrinsic or extrinsic compression secondary to local tumor growth, cervical adenopathy, and/or rapid bleeding. This is a complex and potentially life-threatening oncological emergency. In cases requiring immediate airway relief, surgical airway management remains the mainstay of treatment.^8^

Surgical traineeship is essential but must be safe for patients. In thyroid surgery, surgeon volume correlates with improved clinical and economic outcomes. While the trend in major centers is for thyroidectomy to be performed by head and neck surgeons, in some settings, general surgeons end up performing this procedure. The safety volume threshold is not clearly established. Regarding the specific context of residents, a meta-analysis tends to show a minimal negative impact of their participation in thyroidectomy procedures if they are adequately supervised.^9^

Resolution nº14/2004 of the National Medical Residency Commission (CNRM), dated November 16, 2004, which outlined the contents of the General Surgery Medical Residency Program, established a mandatory one-month internship in Head and Neck Surgery, focusing on three procedures: cervicotomy (access route), tracheostomy, and cervical lymph node biopsy.^10^ However, since 2021, the rotation in this specialty is no longer mandatory in the three-year General Surgery curriculum.^11^

As the topic is relevant, further studies are needed to define the essential contents of a Head and Neck Surgery curriculum for General Surgery residents and fellows in Brazil, to ensure graduates achieve the competency levels necessary for safe patient care. Furthermore, the continuous incorporation of new knowledge into all specialties requires that such content be periodically updated.

Situations that extend beyond primary and secondary care levels, such as staging details and treatment options for various head and neck tumors or complaints requiring specialized examinations, will be experienced during subspecialty professional training. The consensus established in this research proposes comprehensive knowledge of highly prevalent aspects within the specialty, enabling general surgeons to suspect diagnoses, manage low and moderate complexity situations, and appropriately refer patients when necessary.

Regarding the limitations of this study, in the Delphi method, certain biases are particularly expected and should be critically addressed. Participant selection is one of the main sources of bias, since the technique relies heavily on the expertise of the panel. Since the experts were a homogeneous group of specialists, there was some risk of limiting the diversity of opinions and producing an artificially narrow consensus. Another critical issue relates to the agreement criteria used to define consensus. We adopted the arbitrary thresholds of ≥ 85% agreement. It could have introduced bias, either by inflating the perception of consensus or by disregarding relevant nuances of disagreement among experts. Transparence and well-justified definitions of the parameters were employed to minimize methodological subjectivity and ensure greater scientific rigor.

## Conclusion

The consensus established in this study recommends that General Surgery residency and fellowship programs structure their Head and Neck Surgery rotations around clinical scenarios and interventional procedures that surgeons will routinely encounter in practice. Educational emphasis should be placed on mastering management approaches for prevalent pathologies, with balanced allocation of resources between didactic instruction and hands-on surgical training to develop advanced proficiency in addressing common head and neck conditions.

## ORCID ID

Marco Aurélio Vamondes Kulcsar: 0000-0002-4751-0476

Julia Fonseca Ferreira da Silva: 0009-0006-1670-1428

## Authors’ contributions

Mario Augusto Ferrari de Castro: Methodology; data curation.

Rogério Aparecido Dedivitis: Conceptualization; writing-original draft; writing-review & editing.

Fátima Cristina Mendes de Matos: Investigation; formal analysis; writing-review & editing.

Marianne Yumi Nakai: Project administration; methodology.

Leandro Luongo de Matos: Formal analysis.

Marco Aurélio Vamondes Kulcsar: Investigation.

Carlos Takahiro Chone: Supervision; Validation.

Luiz Paulo Kowalski: Supervision; Validation.

Julia Fonseca Ferreira da Silva: Data curation.

Thiago Yuuki Kuroiwa: Data curation.

## Fundings

Nothing to declare.

## Data availability statement

The authors declare that all data are available in repository.

## Declaration of competing interest

The authors declare no conflicts of interest.
